# Textbook Neoadjuvant Outcome—Novel Composite Measure of Oncological Outcomes among Gastric Cancer Patients Undergoing Multimodal Treatment

**DOI:** 10.3390/cancers16091721

**Published:** 2024-04-28

**Authors:** Zuzanna Pelc, Katarzyna Sędłak, Magdalena Leśniewska, Katarzyna Mielniczek, Katarzyna Chawrylak, Magdalena Skórzewska, Tomasz Ciszewski, Joanna Czechowska, Agata Kiszczyńska, Bas P. L. Wijnhoven, Johanna W. Van Sandick, Ines Gockel, Suzanne S. Gisbertz, Guillaume Piessen, Clarisse Eveno, Maria Bencivenga, Giovanni De Manzoni, Gian Luca Baiocchi, Paolo Morgagni, Riccardo Rosati, Uberto Fumagalli Romario, Andrew Davies, Yutaka Endo, Timothy M. Pawlik, Franco Roviello, Christiane Bruns, Wojciech P. Polkowski, Karol Rawicz-Pruszyński

**Affiliations:** 1 Department of Surgical Oncology, Medical University of Lublin, 20079 Lublin, Poland; zuzanna.pelc@umlub.pl (Z.P.); katarzyna.sedlak@umlub.pl (K.S.); 56506@student.umlub.pl (M.L.); 59465@student.umlub.pl (K.M.); 56646@student.umlub.pl (K.C.); magdalena.skorzewska@umlub.pl (M.S.); tomek.ciszewski@wp.pl (T.C.); jczechowska@usk1.pl (J.C.); agata.kiszczynska@wp.pl (A.K.); wojciech.polkowski@umlub.pl (W.P.P.); 2 Department of General Surgery, Erasmus Medical Center, 3015 GD Rotterdam, The Netherlands; b.wijnhoven@erasmusmc.nl; 3 Department of Surgical Oncology, The Netherlands Cancer Institute-Antoni van Leeuwenhoek Hospital, 1066 CX Amsterdam, The Netherlands; j.v.sandick@nki.nl; 4 Department of Visceral, Transplant, Thoracic and Vascular Surgery, University Hospital Leipzig, 04103 Leipzig, Germany; ines.gockel@medizin.uni-leipzig.de; 5 Department of Surgery, Amsterdam UMC location University of Amsterdam, 1007 MB Amsterdam, The Netherlands; s.s.gisbertz@amsterdamumc.nl; 6 Cancer Treatment and Quality of Life, Cancer Center Amsterdam, 1081 HV Amsterdam, The Netherlands; 7 Department of Digestive and Oncological Surgery, University Lille, and Claude Huriez University Hospital, 59000 Lille, France; guillaume.piessen@chu-lille.fr (G.P.); clarisse.eveno@chu-lille.fr (C.E.); 8 Upper G.I. Surgery Division, University of Verona, 37126 Verona, Italy; maria.bencivenga@univr.it (M.B.); giovanni.demanzoni@univr.it (G.D.M.); 9 Department of Clinical and Experimental Sciences, Surgical Clinic, University of Brescia, and Third Division of General Surgery, Spedali Civili di Brescia, 25123 Brescia, Italy; gianluca.baiocchi@unibs.it; 10 Department of General Surgery, Morgagni-Pierantoni Hospital, 47121 Forlì, Italy; morgagni2002@libero.it; 11 Department of Gastrointestinal Surgery, IRCCS San Raffaele Hospital, Vita Salute University, 20132 Milan, Italy; rosati.riccardo@hsr.it; 12 Digestive Surgery, European Institute of Oncology, IRCCS, 20139 Milan, Italy; uberto.fumagalliromario@ieo.it; 13 Department of Upper Gastrointestinal and General Surgery, Guy’s and St Thomas’ Hospital, London SE1 7EH, UK; andrew.davies1@gstt.nhs.uk; 14 Department of Surgery, The Ohio State University Wexner Medical Center and James Comprehensive Cancer Center, Columbus, OH 43210, USA; endo.yutaka@osumc.edu (Y.E.); tim.pawlik@osumc.edu (T.M.P.); 15 Department of Medicine, Surgery, and Neurosciences, University of Siena, 53100 Siena, Italy; franco.roviello@unisi.it; 16 Department of General, Visceral, Cancer and Transplantation Surgery, University Hospital of Cologne, 50937 Cologne, Germany; christiane.bruns@uk-koeln.de

**Keywords:** gastric cancer, neoadjuvant chemotherapy, composite measure, Textbook Neoadjuvant Outcome

## Abstract

**Simple Summary:**

This narrative review aims to present the rationale for the implementation of a novel composite measure, Textbook Neoadjuvant Outcome, among patients with gastric cancer. Textbook Neoadjuvant Outcome integrates five objective and well-established components: Treatment Toxicity, Laboratory Tests, Imaging, Time to Surgery, and Nutrition. It represents a desired, multidisciplinary care and hospitalization of gastric cancer patients undergoing neoadjuvant chemotherapy to identify the treatment- and patient-related data required to establish high-quality oncological care further.

**Abstract:**

The incidence of gastric cancer (GC) is expected to increase to 1.77 million cases by 2040. To improve treatment outcomes, GC patients are increasingly treated with neoadjuvant chemotherapy (NAC) prior to curative-intent resection. Although NAC enhances locoregional control and comprehensive patient care, survival rates remain poor, and further investigations should establish outcomes assessment of current clinical pathways. Individually assessed parameters have served as benchmarks for treatment quality in the past decades. The Outcome4Medicine Consensus Conference underscores the inadequacy of isolated metrics, leading to increased recognition and adoption of composite measures. One of the most simple and comprehensive is the “All or None” method, which refers to an approach where a specific set of criteria must be fulfilled for an individual to achieve the overall measure. This narrative review aims to present the rationale for the implementation of a novel composite measure, Textbook Neoadjuvant Outcome (TNO). TNO integrates five objective and well-established components: Treatment Toxicity, Laboratory Tests, Imaging, Time to Surgery, and Nutrition. It represents a desired, multidisciplinary care and hospitalization of GC patients undergoing NAC to identify the treatment- and patient-related data required to establish high-quality oncological care further. A key strength of this narrative review is the clinical feasibility and research background supporting the implementation of the first and novel composite measure representing the “ideal” and holistic care among patients with locally advanced esophago-gastric junction (EGJ) and GC in the preoperative period after NAC. Further analysis will correlate clinical outcomes with the prognostic factors evaluated within the TNO framework.

## 1. Introduction

With an estimated annual over one million new diagnoses and 770,000 deaths worldwide in 2020, gastric cancer (GC) represents 5.6% of the global cancer incidence and is the fourth-leading cause of all cancer-related deaths [1]. Moreover, considering aging and the growth of the world population, an increase of 62% to 1.77 million cases in GC incidence by 2040 is expected [2]. In locally advanced settings, stomach neoplasms typically require multidisciplinary care. In order to facilitate margin-negative resection and improve survival, esophageal (EC) and GC patients are increasingly treated with neoadjuvant chemotherapy (NAC) prior to curative-intent resection [3].

NAC has evolved into an integral component of multidisciplinary care, contributing to improved locoregional control and comprehensive patient treatment [4]. Almost two decades ago, the MAGIC trial redefined the treatment for locally advanced GC patients by demonstrating a substantial increase in 5-year survival with the addition of perioperative chemotherapy to surgery [5]. Since the MAGIC trial, the role of NAC has been further established with the introduction of the FLOT regimen (Fluorouracil, Leucovorin, Oxaliplatin, and Docetaxel) improving the median overall survival (OS) from 36 to 50 months (HR 0.77; 95% CI: 0.63–0.94) [6]. The role of radiotherapy requires further exploration, as the results from the ARTIST and ARTIST-II trials failed to support its addition in the adjuvant setting, even for patients with lymph-node-positive disease [7,8]. The ongoing phase III TOP GEAR (Trial Of Preoperative therapy for Gastric and Esophagogastric junction AdenocaRcinoma) aims to determine whether neoadjuvant radiotherapy, in combination with perioperative epirubicin/cisplatin/fluorouracil (ECF) chemotherapy, proves superior to perioperative ECF regimen alone [9].

Currently, data supporting the implementation of NAC extend to subsequent gastrointestinal malignancies. Although preoperative radiochemotherapy (41.4 Gy radiotherapy plus carboplatin/paclitaxel, CROSS regimen) has been the gold standard for EC treatment for over a decade, a recent analysis comparing FLOT and CROSS regimens revealed non-inferiority of perioperative chemotherapy, a treatment modality reflecting modern benchmarks of oncologic safety [10]. In the NEO-AEGIS trial, the MAGIC regimen (Epirubicin, Cisplatin, and 5-FU or Capecitabine) was primarily also included in the analysis [11]. Despite the initial assumption of 10% superiority of the CROSS regimen, a futility analysis led to a modification, requiring 5% non-inferiority for perioperative chemotherapy. The results supported clinical equivalence, demonstrating no differences in morbidity, quality of life (QoL), and the 3-year survival probability between CROSS and FLOT/MAGIC arms (HR 1.03, 95% CI 0.77–1.38) [11]. The awaited results of the ESOPEC trial will further evaluate whether FLOT provides survival benefits over CROSS among EC patients [12].

Individually assessed parameters have served as surrogate benchmarks for treatment quality in the past decades [13]. As such, lymph node harvest and R0 resection, surgical quality indicators, impact long-term prognosis and the risk of disease recurrence [14]. While National Comprehensive Cancer Network (NCCN) guidelines recommend the dissection of a minimum of 15 lymph nodes [14], recent data suggest that harvesting at least 23 lymph nodes improves both pathological nodal staging and 5-year survival [15]. However, the optimal nodal yield after NAC is debated, especially considering the stage migration phenomenon, which refers to the relationship between the number of dissected lymph nodes and survival [16]. Corresponding to the evidence from the East [16], the Surveillance, Epidemiology, and End Results (SEER) registry analysis suggested prolonged survival following a greater extent of lymph node dissection [17]. However, Western observations questioned these findings, as more advanced N stage corresponds with tumor burden, and more aggressive lymphadenectomy may result in major surgical trauma [18]. Differences also apply to R0 resection, and despite a unified definition, survival outcomes vary significantly between Eastern and Western centers [19].

Recently, the Outcome4Medicine Consensus Conference, a group dedicated to assessing outcomes in medical interventions, has highlighted the inadequacy of solely examining isolated metrics [13]. This approach fails to fully provide a picture of overall quality and capture the complexity of the clinical scenario. In response to the limitations associated with this singular approach, the adoption of composite measures has gained increased acknowledgment, emphasizing the significance of integrating data from various domains [13]. One of the most simple and comprehensive is the “All or None” method, which refers to an approach where a specific set of criteria must be fulfilled for an individual to be considered as achieving the overall measure [20]. Textbook Outcome (TO) emerged as a comprehensive surgical quality metric, which focuses on desired post-operative outcomes and an ideal hospitalization, representing the multidimensional aspects of the complete surgical pathway. TO not only serves as a prognostic factor but also successfully represents real-world data in a more holistic manner [21,22].

A recently proposed modification of TO among GC patients, Textbook Oncological Outcome (TOO), integrates compliance with perioperative chemotherapy into the standard definition of TO, allowing for a more complex assessment of multimodal oncology care [23]. The analysis of patients with locally advanced GC in Europe revealed a 33% improvement in TO achievement with the implementation of NAC. Although overall TO was achieved in 68.5% of cases, chemotherapy compliance was observed in only 30.8%, resulting in a decreased TOO accomplishment (22.8%). The observed disparity highlights the existing dissonance in evaluating the precisely defined specific components of surgery and NAC, in which the assessment primarily focuses on whether the treatment is administered or not.

Along with the clinical importance of NAC, optimizing QoL and patient-reported outcomes (PROs) during multidisciplinary cancer care is important to patients and healthcare providers. As data on patient-centered outcomes remain scarce [3], designing patient-centered strategies aiming at preventing attrition during NAC remains crucial [24,25].

Consequently, the aim of this narrative review is to present the rationale for the implementation of a novel composite measure, Textbook Neoadjuvant Outcome (TNO). TNO seeks to reflect a desired, multidisciplinary care and hospitalization of selected upper gastrointestinal malignancies. Specifically, we focus on outcomes for NAC among patients with locally advanced esophago-gastric junction (EGJ) cancer and GC undergoing curative-intent treatment to identify the treatment- and patient-related data required to establish high-quality oncological care furthermore.

## 2. Exploring TNO Components

**Figure 1:**
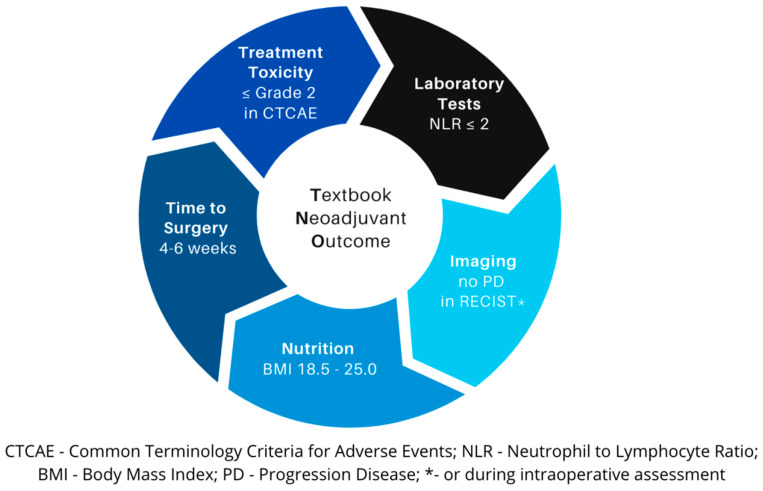
Textbook Neoadjuvant Outcome components.

TNO, designed as an ‘All or None’ composite measure, integrates five objective, well-established, and reproducible components depicted in Figure 1. The fulfillment of each criterion equals TNO achievement, while failure to meet any disqualifies its accomplishment. Each parameter should be assessed during the period between the last NAC cycle and the surgery. The subsequent discussion presents the rationale behind the inclusion of each component in the TNO measure, while the study path is depicted in Supplementary Figure S1.

## 3. Treatment Toxicity

Treatment toxicity, particularly in the context of NAC, can be a critical consideration against preoperative chemotherapy administration [5,26]. Although most studies do not show an increase in postoperative complications, the potential adverse effects (AEs) of NAC may pose challenges to the success of multimodal therapy adherence. As reported in the CRITICS trial, which evaluated the outcomes following NAC in a Western population of locally advanced GC, up to 85% of patients had to discontinue preoperative treatment due to its toxicity, doubling the risk of perioperative complications [27]. Moreover, in the FLOT4-AIO trial, 25% of patients in the FLOT cohort experienced at least one serious adverse event (AE) related to medical or surgical complications [6]. The percentage of patients discontinuing chemotherapy due to FLOT-related side effects surpassed 40%. Notably, among various reasons for perioperative chemotherapy disruption, only 60 (40.5%) patients in the experimental arm completed the planned 8 cycles of systemic treatment out of 148 initially randomized individuals.

While certain reports question the association between NAC toxicity and subsequent treatment outcomes [28,29], there remains an underrepresentation of docetaxel-based regimens. Yet, the FLOT scheme has been recommended as first-line perioperative chemotherapy among locally advanced GC patients in both NCCN and European Society of Medical Oncology (ESMO) guidelines [14,30]. Meanwhile, the most common AEs associated with FLOT are well-documented and include a range of gastrointestinal, hematological, and neurological side effects [6]. Thus, monitoring of toxicities during NAC is crucial to promptly address and manage potential complications, optimize patient well-being, and ensure treatment adherence.

For reporting toxicity, the Common Terminology Criteria for Adverse Events (CTCAE) are used. CTCAE represents the gold standard for classifying and grading the severity of AEs in cancer therapy, clinical trials, and various oncology settings [31]. Employing descriptive terminology, CTCAE provides a grading scale for each adverse event term, offering a systematic and widely accepted approach to reporting and assessing the impact of medical treatments or procedures (Table 1). This comprehensive tool ensures a standardized evaluation of AEs, enhancing communication and facilitating a clearer understanding of their clinical significance.

Addressing and mitigating treatment toxicity is important to optimize the overall success of treatment in the management of GC patients undergoing multimodal therapy. Therefore, we advocate for the incorporation of the latest 5th edition of CTCAE into the assessment of NAC outcomes. Specifically, we propose the inclusion of Grade 1 and Grade 2 toxicity, indicating mild effects that require no or non-urgent medical interventions or therapy, as an additional integral element of the TNO measure.

## 4. Laboratory Tests

Systemic inflammatory response has been established as a significant clinical tool and a prognostic factor associated with adverse outcomes in various malignancies [33,34]. Particularly in GC, a correlation between local inflammation and tumor growth exists [4]. Chronic infection stimulates the development of inflammatory micro-environment and promotes further progression and metastasis of the disease. Recognizing the need for a comprehensive tool including both the impact of inflammation on GC prognosis and a reliable predictive factor, numerous immuno-inflammatory markers have been developed presenting diverse outcomes and distinctive advantages [33,35,36,37]. Among them, arising from complete blood count measurements, the Neutrophil-to-Lymphocyte (NLR) ratio is a well-established prognostic and predictive marker for diverse Asian and European GC populations, evaluated not only before surgery alone, but more significantly, also in the post-NAC setting [35,36,38,39,40]. The preoperative NLR, traditionally recognized as the predictor of long-term outcomes, is increasingly acknowledged for its potential utility in prognosticating short-term outcomes and disease-free survival, particularly in advanced GC patients [41]. Meanwhile, Platelet-to-Lymphocyte Ratio (PLR) [42], Lymphocyte-to-Monocyte Ratio (LMR) [39], Inflammatory Burden Index (IBI) [43], and Prognostic Nutritional Index (PNI) [33] have demonstrated different results in predicting Overall Survival (OS) and risk factors, each presenting unique advantages and limitations in different patient populations (Table 2).

While future considerations may highlight molecular markers like microsatellite instability microsatellite instability (MSI) as a potentially superior predictive tool, its restricted integration into clinical practice due to time and cost limitations contrasts with the simplicity and accessibility of NLR [44]. The cutoff value of NLR ≤ 2 has been suggested as it represents a balance between sensitivity and specificity, providing a practical and clinically relevant threshold for distinguishing between patients with different prognostic outcomes. Therefore, we recommend incorporating NLR ≤ 2 into TNO, serving as a crucial marker for estimating the inflammatory burden in GC patients and its consequential effects on treatment outcomes.

**Table 2:** Summary of Key Inflammatory Markers in GC Patients.

Inflammatory Marker	Study	Population	Results	Distinctive Advantages	Limitations
NLR	Post-hoc exploratory analysis of REAL-2 RCT [35] Retrospective analysis [39]	908 advanced AEG cancer patients undergoing multimodal treatment from UK and Australia 106 locally advanced GC patients	High NLR associated with OS (HR = 1.73, 1.50–2.00) High NLR associated with OS (HR = 1.94, 1.02–3.70)	Predictive factor of short- and long-term outcomes [41], peritoneal and/or metastatic disease [40] Independent prognostic factor in multimodal treatment	Underrepresentation of the population with poor performance status due RCT design Relatively small sample size
PLR	Meta-analysis of 8 studies [42]	4513 GC patients undergoing upfront surgery	High PLR not a reliable predictor for OS (HR = 0.99, 95% CI: 0.9–1.1)	High PLR correlated with a higher risk of LN metastasis and serosal invasion	Not a negative predictor for OS
LMR	Retrospective analysis [39]	106 locally advanced GC patients undergoing NAC	High LMR not a reliable predictor for OS (HR = 0.92, 95% CI: 0.47–1.79)	Reassessment of LMR at post-12-month might be helpful in predicting the long-term survival [45]	Lack of prognostic and predictive role in European population undergoing NAC
IBI	Retrospective analysis [43]	6359 cancer patients	High IBI associated with physical condition, malnutrition, cachexia, and short-term outcomes; independent risk factor (HR = 1.114; 95% CI, 1.072–1.157)	Combined value of NLR and CRP	Asian population, little data regarding GC patients [46]
GPS, mGPS	Retrospective analysis [37]	1710 GC patients undergoing curative or palliative surgery	mGPS associated with postoperative mortality (OR, 1.845; 95% CI, 1.184–2.875)	Indicator of nutritional status, different prognostic value of mGPS depending on tumor stage	Japanese population, prognostic significance of GPS in GC has not been fully investigated
PNI	Two-institutional retrospective analysis [33]	206 AEG and UGC patients undergoing curative-intent surgery	Predictive factor of OS (HR = 8.946) and RFS (HR = 6.416)	Indicator of nutritional status	Asian population, cohort limited to upper GC patients, no assessment after NAC

NLR—Neutrophil-to-Lymphocyte Ratio; PLR—Platelet-to-Lymphocyte Ratio; LMR—Lymphocyte-to-Monocyte Ratio; IBI—Inflammation Burden Index, GPS—Glasgow Prognostic Score; mGPS—modified Glasgow Prognostic Score; PNI—Prognostic Nutritional Index; RCT—Randomized Controlled Trial; AEG—Adenocarcinoma of the EsophaGogastric junction, UGC—Upper Gastric Cancer; GC—Gastric Cancer; NAC—Neoadjuvant Chemotherapy; OS—Overall Survival; HR—Hazard Ratio; CI—Confidence Interval; OR—Odds Ratio; RFS—Recurrence Free Survival; CRP—C-Reactive Protein.

## 5. Radiological Evaluation

Imaging plays a significant role in evaluating tumor response to systemic treatment. Apart from an objective in vivo assessment of disease burden, it allows for determining whether NAC should be pursued, adjusted, or interrupted [47]. According to the ESMO, NCCN, and Japanese Gastric Cancer Treatment guidelines, computed tomography (CT) scanning is routinely used for preoperative staging among GC patients [14,30,48]. Given the increasing role of multimodal therapy in locally advanced settings, the need for a common approach and systematic response assessment in GC is paramount [49]. For over two decades, Response Evaluation Criteria in Solid Tumors (RECIST and RECIST 1.1) have been the standard for response assessment in numerous malignancies, including GC. Objective tumor response for target lesions is determined based on the following criteria: Complete Response (CR): disappearance of all target lesions, with any pathological lymph node diameter < 10 mm in short axis; Partial Response (PR): at least a 30% decrease in the sum of diameters of target lesions, taking as reference the baseline sum diameters; Progressive Disease (PD): at least a 20% increase in the sum of diameters of target lesions, taking as reference the smallest sum on study, as well as appearance of one or more new lesions; Stable Disease (SD): neither sufficient shrinkage to qualify for PR nor sufficient increase to qualify for PD, taking as reference the smallest sum diameters [50]. Since RECIST has been a method of choice in standardized tumor response evaluation among the vast majority of clinical trials, it allows an evidence-based tumor workup in the multimodal setting [51]. Although the criteria are not organ-specific and might not evaluate the critical parameters associated with survival outcomes in specific cancer types and treatments, the RECIST-based endpoint of response rate seems to provide simplicity, availability, cost-effectiveness, and intuitiveness in locally advanced GC [47].

Another limitation of CT arises from the low sensitivity in detecting lymph node metastases, a wide range of sensitivity (23–76%) in diagnosing peritoneal spread, and the preservation of only 1% of unnecessary laparotomies based on restaging CT scans [52]. Possibly, with the advancement of computing power and graphic processing technologies [53], artificial intelligence (AI) techniques demonstrate the potential to enhance CT by providing more accurate staging and restaging of the GC along with improved detection of recurrence and progression of the disease [54]. To address shortcomings of CT, staging laparoscopy (SL) is a recommended complementary diagnostic method in potentially resectable GC, preventing 25% of irrelevant gastrectomies [14,55]. Although SL has limited abdominal cavity exploration, the procedure yield exceeds 36%, with lavage cytology further improving the detection of radiologically occult peritoneal metastasis [56,57]. Moreover, SL maintains its sensitivity independently of lymph node involvement, and its yield has remained consistent over time despite improvements in imaging techniques. A recent Systematic Review indicated a high heterogeneity in procedure technique [55], and there is no consensus on whether to perform repeated SL after NAC as well. However, given the increased rate of minimally invasive gastrectomy in the West, performing laparoscopy with intraoperative decision to pursue with curative intent gastrectomy, may condition further assessment of surgical textbook outcomes. SL could be considered as a “bridge to cross to TO”, facilitating the transition from neoadjuvant assessment to the comprehensive evaluation of surgical outcomes.

Despite the limited prognostic value of strict radiological downstaging when comparing baseline and post-NAC CT among locally advanced GC patients from the West [58], we suggest including no PD as a radiological component of TNO. An acceptable alternative to restaging CT is the intraoperative assessment of disease burden at the initial stage of surgical procedure, providing a complementary and potentially more accurate evaluation of treatment response.

## 6. Nutrition

Almost 20% of cancer-related deaths are caused by malnutrition rather than malignancy itself, with up to 40% of malnourished patients being misdiagnosed [59,60]. Gastrointestinal cancer patients are especially at high risk of developing malnutrition, with prevalence rates ranging from 30% to 80% [61].

The European Society for Clinical Nutrition and Metabolism (ESPEN) recommends Nutrition Risk Screening-2002 (NRS-2002) and the Malnutrition Universal Screening Tool (MUST) for malnutritional risk screening in the general population, and Mini Nutritional Assessment (MNA) for geriatric patients [62]. However, the application of these tools is confined to prescreening and identifying patients “at nutritional risk”, a condition associated with increased morbidity and mortality. To confirm the malnutrition diagnosis and perform a nutritional status assessment, ESPEN specifies the fulfillment of one of the following criteria: body mass index (BMI) < 18.5 kg/m^2^, reduced BMI accompanied by weight loss, or decreased fat-free mass index (FFMI) [62]. Although weight loss within a defined timeframe is a straightforward approach in research and routine oncology practice, it is considered unimodal and oversimplified, insufficient to solely use to diagnose malnutrition [63]. The exclusive assessment of BMI holds superior diagnostic value compared to weight loss, with BMI being the only prognostic factor among ESPEN criteria for patients with gastrointestinal cancers [62,64].

Nonetheless, it is crucial to recognize the limitations within this approach as malnutrition occurs independently of initial body weight and obesity does not exclude the concurrent diagnosis of sarcopenia or cachexia [63]. While the negative impact of underweight as a prognostic factor has been well-established, the landscape of overweight remains more complex and inconclusive [61,63]. Apart from the “obesity paradox”, the phenomenon of lower mortality associated with increased weight is explained by potential selection bias; even more than 60% of gastrointestinal cancer patients with excessive body mass will develop malnutrition [63,65]. This group of patients often remains overlooked in routine nutritional screening; meanwhile, silently developed cachexia or sarcopenia leads to poor prognosis and decreased survival [66].

Patients undergoing NAC represent another distinct group requiring a specific nutritional approach. The preoperative period remains clinically significant for the implementation of prehabilitation—a comprehensive approach that combines physical and nutritional therapy [67]. The main objective is to optimize patients’ fitness and prepare them for metabolic stress and surgical trauma, thereby enhancing adherence to perioperative chemotherapy. A systematic review of randomized controlled trials of prehabilitation incorporated a range of exercise training and psychological and nutritional interventions with diverse effectiveness [68]. Despite the absence of a standardized protocol, this multifaceted approach proves non-inferior to the established standard of care and merits consideration as an integral component of contemporary patient-centered healthcare.

Another interesting concept is frailty, a multidimensional syndrome often accompanied by malnutrition and sarcopenia, which represents decreased physiologic reserve and ability to recover from different stressors [69]. Frailty is a dynamic state that mirrors biological age, influenced by conditions such as comorbidities or social determinants of health. Frail GC patients are at significantly higher risk of mortality (6.83 vs. 3.50%), morbidity (3.42 vs. 0.94%), and prolonged length of stay (16.7 vs. 12.0 days) when compared with non-frail individuals [70]. While the concept is promising, the National Surgical Quality Improvement Program (NSQIP), established by the American College of Surgeons, has emphasized the need for additional efforts in defining and assessing frailty [69,71].

Specific nutritional strategies to overcome body composition deficiencies are recommended, aligning with Enhanced Recovery After Surgery (ERAS) principles [72]. Unlike conventional perioperative approaches, ERAS integrates cutting-edge techniques in anesthesiology, pain management, nutrition, psychology, and surgery, combining them with traditional methods to enhance postoperative recovery. It is estimated that even 65% of surgical patients present some degree of malnutrition, and adherence to ERAS protocol improved 5-year survival in colorectal cancer patients correlated with early nutritional delivery in the postoperative period [73]. However, its adoption in gastrointestinal surgery remains underrepresented, largely due to distinct traditional post-gastrectomy management practices [74]. Additionally, the impact of ERAS is evaluated after the surgery, and the tool assessing patients’ condition in the post-NAC setting is lacking.

Given that both excessive- and underweight status not only enhance treatment-related toxicity but also significantly increase mortality rates, it is crucial to assess the nutritional status of patients with gastrointestinal malignancies at the early stages of oncologic treatment in a simple and widely accepted manner [66,75,76]. Therefore, evaluating and addressing the nutritional status of patients within TNO through BMI seems beneficial and an applicable method to optimize treatment outcomes, including NAC tolerability. Based on the World Health Organization (WHO) [77] we adopt BMI ranging from 18.5 to 24.9 kg/m^2^ as one of the components of TNO.

## 7. Time to Surgery

Since NAC potentially allows for primary tumor downstaging and may contribute to the clearance of clinically occult nodal- and micro-metastases, it is important to determine factors increasing the likelihood of pathologic response (PR), ultimately resulting in improved OS [6]. Association between completion of preoperative therapy and time to surgery (TTS) has been suggested as one of the factors influencing the PR in several gastrointestinal malignancies. While a prolonged (6–8 week) interval between neoadjuvant radiotherapy and surgery showed increased tumor downstaging with no detrimental effect on toxicity in rectal cancer [78,79], breast cancer patients undergoing surgery within three weeks after completion of NAC experienced improved survival outcomes, with TTS being an independent prognostic factor, even among patients with complete PR [80]. However, in EC patients undergoing preoperative radiochemotherapy, TTS exceeding 6 weeks negatively impacted survival with no significant improvement in PR, tumor regression, or radicality of resection [81].

A recent meta-analysis of retrospective observational studies that included 1171 patients undergoing gastrectomy after NAC within three timeframes—4–6 weeks, <4 weeks, and >6 weeks—reported comparable outcomes in terms of complete PR, R0 resection rate, and the incidence of serious postoperative complications, as well as 3-year progression-free survival (PFS) and OS [82]. A subsequent meta-analysis compared patient outcomes between TTS within 4–6 weeks and 4–6 weeks after NAC completion among patients with locally advanced GC [83]. Pooled data were not associated with significant differences in major and complete PR rates, ypN0, postoperative complications, R0 resection rates, and operative time between groups of longer TTS and shorter TTS. However, when taking into account the Western population, the highest rate of major PR was achieved in patients undergoing gastrectomy within 4 weeks after NAC completion compared with individuals receiving surgical treatment within 4–6 weeks or later [84]. Furthermore, shorter TTS was associated with similar postoperative morbidity and mortality. At the same time, additional medical optimization in preparation for surgery may offer benefits without impacting outcomes or nodal upstaging [85]. Although large-scale prospective randomized controlled trials are warranted to establish optimal TTS among locally advanced GC patients undergoing multimodal treatment, we suggest a 4–6 week time interval to gastrectomy after NAC as a TNO component.

## 8. Systemic Therapy

In recent years, the treatment landscape of GC has undergone significant evolution, primarily driven by the introduction of novel immunotherapies and targeted treatment options applicable across diverse disease stages [4,30]. Following these advancements, a noticeable shift has been observed towards an individualized approach and biomarker-tailored treatments, particularly in the metastatic setting. However, for locally advanced disease, perioperative chemotherapy and curatively-intended surgery remain the cornerstone of sustainable cancer treatment [14].

The administration of chemotherapy before surgical management offers both apparent and more subtle benefits [44]. Chemotherapeutic agents are delivered to the primary site before surgical vasculature disruption more efficiently. Apart from downstaging the primary tumor and increasing the likelihood of radical resection, NAC appears to be better tolerated compared with adjuvant treatment [6]. Although limited by tissue heterogeneity, a hallmark of GC response to NAC provides an insight into tumor chemosensitivity [86,87]. The transition of recent findings in molecular biology into efficient therapeutic solutions remains one of the greatest challenges in the multimodal treatment of GC patients [4]. For example, results of the ToGA trial incorporated into clinical practice trastuzumab, a monoclonal antibody targeting Human Epidermal Receptor (HER2) over a decade ago [88]. The addition of trastuzumab to platinum-based doublet first-line palliative chemotherapy in GC patients with HER2 overexpression was associated with a decrease in the risk of death by 26%. Another milestone in the multimodal therapy of GC was established along with the results of the FLOT4-AIO trial, incorporating a docetaxel-based regimen as the perioperative treatment of choice for medically fit patients [89]. However, the impact of taxanes has been debated due to potential toxicity and uncertain clinical benefits in the geriatric population [30,89].

Recently, the results of the GASTFOX-PRODIGE 51 trial [90] were presented at the ESMO 2023 Congress. The study’s objective was to verify the benefit of integrating docetaxel into FOLFOX (modified FLOT = TFOT) in previously untreated advanced GC patients. In the TFOT group, an improved OS (HR = 0.82, 95% CI 0.68–0.99), PFS (median: 7.59 vs. 5.98, *p* = 0.007), and objective response rate (ORR, 66.2% vs. 57.5%, *p* = 0.04) were observed compared with a control group receiving FOLFOX. These findings further strengthen the significance of incorporating docetaxel into first-line chemotherapeutic regimens in advanced stages of GC.

However, up to 10% of locally advanced GC tumors present high MSI and programmed death-ligand 1 (PD-L1) expression, being the potential candidate for perioperative immune-checkpoint inhibitors (ICI) therapy. In the third interim analysis of the KEYNOTE-585 trial, the addition of pembrolizumab to chemotherapy demonstrated an increase in pathologic complete response (12.9% vs. 2%; *p* < 0.0001) while concurrently showing a statistically insignificant improvement in event-free survival (EFS, median 44.4 months vs. 25.3 months; HR 0.81; 95% CI 0.67–0.99; *p* = 0.0198) [91]. In a similar design, the MATTERHORN trial randomized patients to receive perioperative FLOT with placebo or FLOT plus durvalumab (anti-PD-L1 antibody), revealing a higher pathologic complete response rate in the group treated with the addition of durvalumab [92]. The percentage of patients undergoing surgery and achieving R0 resection was comparable between the two groups. The investigation is currently ongoing, focusing on the primary endpoint of EFS. It is crucial to highlight that patients included in these studies were biomarker unselected, therefore the survival benefit of perioperative immunotherapy is uncertain.

One of the main limitations of systemic chemotherapy is restricted penetration into the peritoneum, the most frequent location of metastatic disease and site of recurrence. The peritoneal dissemination may be diagnosed even in 40% of cases, most frequently during diagnostic laparoscopy, and reduce the median survival from 14 to 4 months [93,94]. The revolution in the treatment of peritoneal metastases was introduced by Paul Sugarbaker in 1989, proposing the Hyperthermic IntraPeritoneal Chemotherapy (HIPEC) method [95]. Despite evidence suggesting its effectiveness, the lack of high-quality evidence from randomized controlled trials restricts the widespread adoption of this therapy in GC patients, confining it to experimental settings only [96]. Among prophylactic, palliative, and pre- and postoperative indications for HIPEC, a group of patients undergoing NAC at high risk of peritoneal dissemination require special consideration, given the potential challenges in achieving curative-intent surgery. The therapeutic value of adjuvant HIPEC in patients with peritoneal metastases after NAC was suggested by the GASTRICHIP trial results [97]. The interim safety analysis confirmed that intraperitoneal perfusion of oxaliplatin with concurrent intravenous 5-FU administration results in equal morbidity when compared to cytoreductive surgery alone [98]. Although current data have not impacted actual state-of-the-art, results of the PERISCOPE II trial comparing standard-of-care systemic chemotherapy versus HIPEC [99] and the PREVENT trial assessing prophylactic use of HIPEC along with FLOT chemotherapy are anticipated [100].

Although identifying and mitigating risk factors for preoperative chemotherapy compliance and effectiveness is critical for improving outcomes of treatment, the proposed methodology for TNO implantation has certain limitations. First, while the TNO embraces various aspects of GC management, it may suffer from a lack of specificity regarding the methodology employed for data collection and analysis. Additionally, some rationales for TNO components such as time to surgery or nutrition rely on retrospective data, as there is no clear consensus or guidelines recommendations. Furthermore, the discussion on numerous evolving paradigms in GC treatment is provided, although without assessing the impact of these advancements on the proposed composite measure. However, it is important to note that the proposed version of TNO was developed through multi-institutional expert discussions, indicating a collaborative effort to address these limitations. Additionally, TNO will be further evaluated in prospective settings, which is crucial for validating its effectiveness and reliability in assessing outcomes of GC patients undergoing neoadjuvant therapy.

## 9. Conclusions

Despite the recent advancements in GC management and ongoing exploration of preoperative therapy, further investigations should help to establish outcomes assessment of current clinical pathways. A key strength of this narrative review is the demonstrated feasibility and the provided research background supporting the implementation of the first and novel composite measure representing the “ideal” and holistic care among patients with locally advanced GEJ and GC in the preoperative period. TNO might be useful for identifying and addressing specific areas of improvement, specifically chemotherapy compliance, to enhance the overall quality of care delivered to GC patients undergoing curatively-intended multimodal treatment. Further, in the ongoing discourse on the reevaluation of TO in GC surgery [19], we anticipate an evolution of the TNO scoring system due to continuous preoperative therapy investigations [9,101]. Moreover, it is necessary to conduct further analysis that will correlate clinical outcomes with the prognostic factors evaluated within the TNO framework.

## Figures and Tables

**Table 1:** Overview of TNO Components.

TNO Category	Description		
Imaging **			
Complete Response *	**Disappearance of all target lesions, with any pathological lymph nodes diameter < 10 mm in short axis.**		
Partial Response *	**At least a 30% decrease in the sum of diameters of target lesions, taking as reference the baseline sum diameters.**		
Progressive Disease	At least a 20% increase in the sum of diameters of target lesions, taking as reference the smallest sum on study, as well as appearance of one or more new lesions.		
Stable Disease *	**Neither sufficient shrinkage to qualify for PR nor sufficient increase to qualify for PD, taking as reference the smallest sum diameter.**		
Timing to Surgery			
≤4 weeks	32% of mPR in European cohort [32]; twofold higher odds for achievement of mPR (OR 2.09; 95% CI 1.01–4.34, *p* = 0.047).		
4–6 weeks *	**Highest rate of ypT3-4 tumors (67.5%) and any postoperative complications (44.9%).**		
>6 weeks	Highest rate of lymphovascular invasion and ypN+ (62.5%), lowest rate of NAC completion (84.7%).		
Nutrition BMI < 18.5 kg/m^2^ **BMI 18.5–25 kg/m^2^** * BMI > 25 kg/m^2^ 25–29.9 kg/m^2^ >30 kg/m^2^	Underweight **Normal weight** Overweight Pre-obesity Obesity	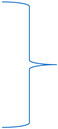	Among ESPEN criteria, BMI is the only one associated with prognosis; nutritional status deterioration may occur independently of body weight thus it should be assessed at early stage of oncologic treatment.
Laboratory Tests NLR ≤ 2 * NLR > 2	**Low NLR; favorable prognosis, increased OS.** High NLR; decreased OS and PFS.		
Treatment Toxicity CTCAE v.5			
Grade 1 *	**Mild; no intervention needed, asymptomatic or mild symptoms.**		
Grade 2 *	**Moderate; requires minimal intervention; affects age-appropriate instrumental ADL.**		
Grade 3	Severe or medically significant; not immediately life-threatening; requires hospitalization; affects self-care ADL.		
Grade 4	Life-threatening consequences; requires urgent intervention.		
Grade 5	Death related to adverse event.		

TNO—Textbook Neoadjuvant Outcome; *—TNO Component; **—according to Computed Tomography or intraoperative assessment; mPR—major Pathological Response; OR—Odds Ratio; CI—Confidence Interval; ypT—post-neoadjuvant pathological tumor stage; ypN—post-neoadjuvant pathological nodal stage; BMI—Body Mass Index; ESPEN—European Society for Clinical Nutrition and Metabolism; NLR—Neutrophil-to-Lymphocyes Ratio; OS—Overall Survival; PFS—Progression Free Survival, CTCAE—Common Terminology Criteria for Adverse Events; ADL—Activities of Daily Living. Bold characteristics indicate TNO components

## Supplementary Materials

The following supporting information can be downloaded at: https://www.mdpi.com/article/10.3390/cancers16091721/s1. Figure S1: Flow chart of study path.
