# Supplementary figures and images for: Textbook Neoadjuvant Outcome—Novel Composite Measure of Oncological Outcomes among Gastric Cancer Patients Undergoing Multimodal Treatment
Source: Cancers (Basel). 2024 Apr 28;16(9):1721. doi: 10.3390/cancers16091721 (PMC11083243; doi:10.3390/cancers16091721)

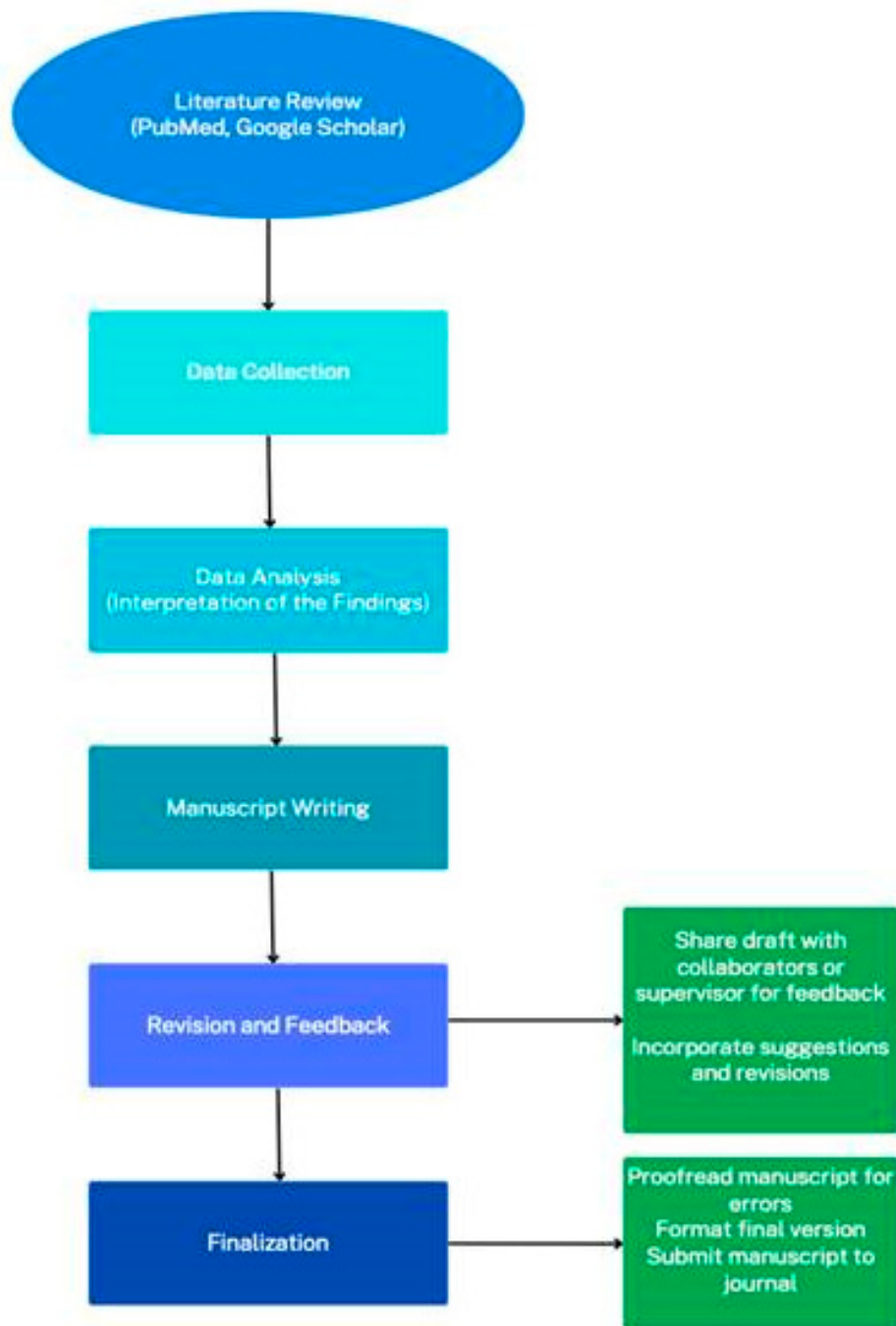

**Figure S1.** Flowchart of study path.

Supplement: Supplementary file 1 [file cancers-16-01721-s001.zip › cancers-2976779-supplementary.pdf]
